# Who puts the “support” in supportive housing? The relationship between housing staff support and resident experiences, and the potential moderating role of self‐determination

**DOI:** 10.1002/ajcp.70023

**Published:** 2025-10-05

**Authors:** Kenna E. Dickard, Greg Townley

**Affiliations:** ^1^ Department of Psychology Portland State University Portland Oregon USA

**Keywords:** community mental health, self‐determination, serious mental illness, supportive housing, well‐being

## Abstract

The provision of residential and community‐based services for individuals with serious mental illness (SMI) has become increasingly important following the deinstitutionalization movement. Much of the existing research on supportive housing focuses on housing outcomes rather than exploring how the program helps its residents thrive in the broader community. This study draws upon data collected from 176 people with SMI residing in 16 supportive housing locations in Portland, Oregon. Analyses explore how housing staff support relates to residents' loneliness (interpersonal level), residential satisfaction (housing and neighborhood level), and sense of community (community level). Staff support was found to be related to lower levels of loneliness, higher residential satisfaction, and a higher sense of community. Self‐determination was considered as a moderator to understand the role of residents' agency in the relationships between staff support and resident experiences. Self‐determination moderated the relationship between staff support and residential satisfaction for those with moderate to low self‐determination, but not for individuals with high self‐determination. In contrast, staff support was associated with decreased loneliness and increased sense of community regardless of self‐determination. This study has implications for policymakers, researchers, and interventionists, expanding upon the limited body of research on staff support and the experiences of residents in a supportive housing environment.

## Highlights

- Staff support favorably impacts resident experiences.
- Staff support increased residential satisfaction for those with lower levels of self‐determination.
- Staff support reduces loneliness in residents with SMI.

The landscape of mental health care in the United States was revolutionized in the 1960s during the deinstitutionalization movement. Before this shift, individuals with serious mental illnesses (SMI) were primarily treated in institutionalized settings which often provided substandard care and failed to emphasize reintegration into society. The Community Mental Health Act of 1963 was a landmark policy that introduced community‐based mental health centers as an alternative to hospitalization. This act aimed to provide accessible mental health services while allowing individuals to live and work within their communities. By 1980, over 700 community mental health centers had been established, serving millions of individuals annually (Carling, 1995). Despite this progress, a gap remained for those with SMI who required more intensive, long‐term support (Hennessy & Greenberg, 1994).

## SUPPORTIVE HOUSING

Supportive housing emerged as a critical solution to this gap, offering affordable, independent housing combined with flexible, person‐centered community‐based mental health services (Rudkin, 2003). Supportive housing encompasses a range of models, including scattered‐site supportive housing and congregate supportive housing, which differ in their structure and level of support (Carling, 1993; Spector et al., 2020). Scattered‐site supportive housing typically provides rental assistance for individuals to live in market‐rate apartments dispersed throughout the community, with support services offered off‐site or on an as‐needed basis. In contrast, congregate supportive housing, also referred to as single‐site supportive housing, consists of apartment complexes where most or all residents are individuals with serious mental illnesses (SMI). In this type of housing, on‐site staff provide varying levels of support, such as assistance with connecting to services, group activities, and peer‐led programming. This study focuses on congregate supportive housing, whereby residents have access to on‐site staff who assist with practical concerns, such as navigating paperwork, managing neighbor disputes, and connecting to mental health services, while maintaining independent living arrangements. By situating this study within congregate independent supportive housing, we aim to address gaps in the literature regarding how housing staff support is associated with residents' experiences of loneliness, residential satisfaction, and sense of community.

Existing research underscores the association between supportive housing and clinical outcomes. For instance, Leff and Trieman (2000) found that individuals in supportive housing experienced reduced psychiatric symptoms, improved social behaviors, and expanded social networks compared to those who remained hospitalized. Aubry's (2020) meta‐analysis further supports the effectiveness of permanent supportive housing in enhancing housing stability without adverse effects on social and health outcomes.

Research has also begun to explore the broader impacts of supportive housing beyond housing stability, highlighting its role in fostering community integration, recovery, and well‐being. For example, Ornelas et al. (2014) emphasize the importance of social connections in recovery, showing that supportive housing can enhance residents' sense of belonging and participation in community life when tailored social support systems are in place. Similarly, Eklund et al. (2017) found that individuals with serious mental illnesses living in supported housing reported higher levels of health, quality of life, and recovery than individuals in ordinary housing, possibly due to higher levels of access to social interaction. Spector et al. (2020) further demonstrated that housing stability, coupled with access to flexible and individualized support, contributed to residents' improved mental health and reduced experiences of loneliness. These studies underscore the need for supportive housing programs to prioritize relational and community‐based supports, as they are integral to residents' broader recovery and well‐being.

This study builds on these findings by examining housing staff support as a key factor in fostering positive experiences within a congregate supportive housing context. Adopting an ecological systems approach (Kloos & Shah, 2009), this study investigates resident experiences at an interpersonal, housing/neighborhood, and community level. It also considers the role of self‐determination in the relationship between staff support and resident experiences, emphasizing the active participation of residents in their care.

## ROLE OF STAFF

The shift from a symptom‐focused approach to a whole‐person approach in mental healthcare led to the emergence of nonclinical roles such as peer support specialists, health and wellness coaches, and case managers (Myrick & del Vecchio, 2016). These mental health professionals assist residents with more than just symptom management, offering support with social skills, navigating community resources, and increasing physical activity.

Research on the impact of community‐based staff is promising yet limited. Chen (2016) used qualitative techniques to explore staff practices in a clubhouse setting and found that social relationships with staff members fostered goal completion in clients. Moreover, they found that negative staff relationships were one reason that clients stopped attending programming at the clubhouse. Another qualitative study explored beneficial relationships in supportive housing from the staff's perspective (Lindvig et al., 2020). The overarching theme of this study was the importance of reciprocity in resident–staff relationships. Researchers found that contributions from both residents and staff, and a recognition of these contributions, were essential to progress made on mutual goals. Moreover, closer resident–staff relationships led to higher motivation for staff to find creative solutions to the resident's complex resource needs, indicating the important role the resident plays in the staff's ability and willingness to support them.

One quantitative study explored staff activity in supportive housing and found that a higher proportion of staff in relation to residents was not a positive contributor to residents' experiences (Felce et al., 2002). This finding aligns with previous research that indicates that simply increasing staff does not positively impact the residents' outcomes (Felce, 1998). Felce and colleagues (2002) did find, however, that having staff with more experience was associated with residents receiving more attention and assistance. This study indicates the importance of retaining long‐term staff in this environment. The consistency of staff also provides greater opportunities for residents to develop trust and familiarity with their support providers.

## LONELINESS

To examine the role that on‐site support staff may play in residents' well‐being, the current study explores interpersonal, housing/neighborhood, and community experiences, which will be reviewed in the following three sections. First, loneliness has been explored extensively among individuals with SMI due to its high prevalence in this population and its connection to negative health outcomes (Trémeau et al., 2016). Loneliness is an emotional state that reflects unpleasant subjective experiences due to perceived social isolation (Weiss, 1987). Over three‐fourths of adults with SMI report loneliness, compared to one‐fourth of the general population (Perese & Wolf, 2005). Contributing factors include smaller social networks, social exclusion due to stigma, and symptoms like depression and paranoia leading to isolation (Chen, 2016; Schwartz & Gronemann, 2009).

Research highlights the importance of addressing loneliness in supportive housing environments. Weiner et al. (2009) found higher feelings of isolation and loneliness in supportive housing residents, particularly in the first 3 months. Similarly, Nagata et al. (2022) reported higher loneliness immediately after moving in, which decreased with longer residence, suggesting that housing stability reduces loneliness. Qualitative research by Piat et al. (2017) indicated that maintaining social connections and family involvement are crucial for managing loneliness, with staff efforts to provide activities and community engagement being appreciated by residents.

Limited research exists on staff's ability to reduce loneliness in supportive housing, though studies in other settings suggest potential benefits. One study exploring loneliness in an aged care facility found that residents who required nurses to support them with daily activities reported lower levels of loneliness after controlling for social network quality (Franklin & Tranter, 2006). Another qualitative study reported staff support as a common theme when exploring factors that decreased loneliness among residents in a managed care facility (Ballin & Balandin, 2007). These findings illustrate the potential role of staff support in decreasing loneliness among residents, although research has not been conducted specifically among individuals with SMI living in community settings.

## RESIDENTIAL SATISFACTION

Another commonly explored construct in the mental health and housing literature is residential satisfaction. Residential satisfaction is a multidimensional construct encompassing physical housing and neighborhood characteristics, access to resources, and social support and safety perceptions. Studies reveal that individuals with SMI often live in more distressed and unstable neighborhoods compared to those with developmental disabilities, impacting their neighborhood satisfaction and social contact (Kruger et al., 2007; Wong & Stanhope, 2009). Additionally, perceived social support and connectedness are crucial for residential satisfaction. Individuals in supportive housing report less social contact with neighbors and lower life satisfaction than the broader community (Aubry & Myner, 1996). Fostering social and community integration is vital, as positive social interactions predict improved well‐being (Kloos & Shah, 2009).

Despite these concerning findings comparing individuals with SMI to other populations, research within this community shows promise for residential satisfaction in supportive housing. Research has consistently demonstrated that individuals in supportive housing reported higher residential satisfaction than those in more restrictive environments such as residential treatment centers (Newman, 2001; Tsemberis et al., 2003). While these findings show a great deal of progress in the field, there is still a long way to go to close the gap in residential satisfaction between individuals with SMI and the broader community.

Support from on‐site staff is one unique offering of supportive housing that could play a role in closing this gap. Patterson et al. (2014) compared participants in supportive housing with a treatment‐as‐usual group and found that individuals in supportive housing had improved psychiatric outcomes. The authors attributed these outcomes to on‐site staff support, specifically staff meeting residents in their neighborhoods to assist with community integration. Moreover, Piat et al.'s (2009) qualitative study with residents of a supportive housing program found that for some participants, a sense of security was associated with the staff's physical presence at the housing site.

## SENSE OF COMMUNITY

The final construct explored in this study is the individuals' sense of community (SOC). SOC is one of the most widely explored constructs in Community Psychology and is associated with many favorable outcomes for individuals with SMI (Townley & Kloos, 2009). Sarason (1974) first defined this concept as a psychological sense of community (PSOC) and argued its relevance in mainstream psychology as an integral part of community and collective well‐being. SOC is related to various other individual outcomes, including safety (Ziersch et al., 2005); length of residence (Perkins, 2009); loneliness (Pretty et al., 1996); and severity of mental health symptoms (Ellaway et al., 2001). Research on supportive housing staff's role in fostering SOC is limited but promising. Forenza and Lardier (2017) found that staff facilitate community connections and organize activities that promote SOC. McCarthy and Nelson (1993) reported that staff support is essential for community integration, helping residents with problem‐solving and resource connections.

## SELF‐DETERMINATION

The evidence outlined above suggests that staff support may be associated with favorable outcomes for residents' loneliness, residential satisfaction, and sense of community. However, additional factors may influence the relationship between staff support and these three constructs. One such factor is the resident's self‐determination, a concept developed by Deci and Ryan (1985). Self‐determination theory (SDT) highlights the importance of autonomy, competence, and relatedness in driving motivation and behavior. Individuals with high self‐determination are motivated to control their lives, develop mastery, and connect with others, which can enhance their engagement in treatment and quality of life (Jochems et al., 2017; Mattner et al., 2017).

Self‐determination is crucial in supportive housing, where fostering independence is a core goal. Studies indicate that quality staff relationships enhance residents' autonomy and well‐being (Pejlert et al., 1999; Peterson et al., 2021). Conversely, perceived negative staff support can reduce autonomy (Welch & Cleak, 2018). Staff find it easier to support residents who take ownership of their issues, suggesting that high self‐determination can lead to better staff‐resident interactions and outcomes (Lindvig et al., 2020). In the context of supportive housing, staff may serve as a bridge to connection—particularly for residents experiencing social isolation. However, we propose that the relationship between staff support and the three variables may be shaped by individual differences in self‐determination. Residents with higher self‐determination may be more likely to engage in reciprocal and meaningful interactions with staff, making staff support more impactful in reducing loneliness, increasing residential satisfaction, and sense of community. While individuals with lower self‐determination may rely on staff support more heavily, such support may be experienced as more instrumental or obligatory, limiting its effectiveness.

The above findings indicate the need to consider self‐determination as a moderator in the relationship between staff support and resident experiences of loneliness, residential satisfaction, and sense of community. While self‐determination has not been considered a moderator in the supportive housing literature, it has been examined as a moderator in other research among individuals with SMI to examine depressive symptoms and suicidality (Bamonti et al., 2014). Another study examining an alcohol intervention for college students found that self‐determination moderated the association between intervention and outcome, such that for highly self‐determined individuals, the intervention was more effective (Neighbors et al., 2006). These findings suggest that self‐determination may be a useful moderator in evaluating programs for individuals with serious mental illnesses.

## STUDY PURPOSE, RESEARCH QUESTIONS, AND HYPOTHESES

A foundational assertion of supportive housing is that recovery is possible for individuals with serious mental illnesses. With the appropriate amount of structure and support, individuals with SMI can live and contribute to society in meaningful ways. Despite efforts to foster independence and thriving, residents of these programs often continue to face challenges in their daily experiences compared to the general population. This has led to criticisms that supportive housing may not always provide the types or levels of support residents need to thrive independently in their communities. These concerns are understandable, given that research on resident experiences—such as feelings of connection, satisfaction with housing, and social inclusion—is still limited. Further research is needed to examine how specific aspects of supportive housing are associated with these experiences. In particular, identifying which features of supportive housing best support positive resident experiences can help guide funding decisions and inform future program development.

This study considers how housing staff support relates to residents' experiences of loneliness, residential satisfaction, and sense of community, each chosen to reflect different aspects of resident experiences—interpersonal, housing, and broader community—within an ecological framework. Loneliness captures the interpersonal experiences, reflecting the degree of social isolation or connectedness residents experience, which has significant implications for mental health and overall well‐being. Residential satisfaction represents the housing experience, assessing how content residents feel with their immediate living environment, a key predictor of stability and quality of life in supportive housing contexts. Finally, sense of community addresses the broader community experience, reflecting residents' feelings of belonging and connectedness within their neighborhoods.

Together, these constructs align with an ecological systems perspective, which emphasizes the interdependence between individuals and their environments (Kloos & Shah, 2009). By examining residents' experiences through these interconnected constructs, this study provides a nuanced understanding of how supportive housing and housing staff support influence residents' experiences, offering insight into the structural and relational factors that foster thriving and recovery in supportive housing programs. In recognizing the residents as active contributors to their experiences in supportive housing, this study also considers how self‐determination may influence this association.

**Research Question 1:** Does housing staff support in a supportive housing environment relate to residents' loneliness, residential satisfaction, and sense of community?

Hypothesis 1a Stronger housing staff support will be associated with lower loneliness

Hypothesis 1b Stronger housing staff support will be associated with higher residential satisfaction

Hypothesis 1c Stronger housing staff support will be associated with higher sense of community

**Research Question 2**: How does self‐determination affect the strength of the association between housing staff support and residents' loneliness, residential satisfaction, and sense of community?

Hypothesis 2a The relationship between housing staff support and loneliness will be moderated by self‐determination, such that for residents with higher self‐determination, the association between housing staff support and loneliness will be more strongly negative relative to those with lower self‐determination

Hypothesis 2b The relationship between housing staff support and residential satisfaction will be moderated by self‐determination, such that for residents with higher self‐determination, the association between housing staff support and residential satisfaction will be more strongly positive relative to those with lower self‐determination

Hypothesis 2c The relationship between housing staff support and sense of community will be moderated by self‐determination, such that for residents with higher self‐determination, the association between housing staff support and SOC will be more strongly positive relative to those with lower self‐determination.

## METHODS

### Participants

This study examines data collected in 2014 from 176 residents living in 16 independent supportive housing sites operated by a single housing provider in Portland, Oregon. The level of support provided across the sites varied, but all sites had on‐site housing managers who assisted residents with tasks such as connecting to services, resolving neighbor disputes, completing paperwork, and addressing household issues. Some sites also offered peer support providers and optional on‐site group activities. All programming and engagement with staff were optional, allowing residents to choose the level of support they wished to receive. Each unit was an independent living unit, where residents lived alone, held their own leases, and accessed support as needed either through the housing staff or the associated mental health center. The supportive services available included housing retention services, case management, skills training, and other mental health supports. Recruitment for the study involved sending letters to all 324 eligible residents, advertising a project focused on resident perspectives on housing and community experiences. Of the eligible participants, 54% completed the study.

Participant ages ranged from 22 to 72 (*M* = 50.05, SD = 9.66). Just over half of participants (56%) identified as male, 43% as female, and 1% as nonbinary. The majority of participants were white (68%), with the remaining participants identifying as Black (24%), Latino (2%), Native American (2%), Asian (1%), and other/race not listed (3%). Bipolar Disorder (28%) represents the most common primary diagnosis, followed by schizophrenia‐spectrum disorder (25%), major depression (19%), and anxiety (18%). On average, participants had lived in the current housing for 5.6 years. Rates of engagement with mental health services were high, with an average of 81% of participants taking psychiatric medication and 70% participating in therapy in the last 6 months. Most participants (76%) reported a history of homelessness, and 20% reported a psychiatric hospitalization since moving into housing. Participants reported a mean of three lifetime episodes of homelessness, with the average age of first becoming homeless at 32. Most participants received social services support, with 94% receiving food stamps, 93% receiving Medicaid, and 84% receiving a housing subsidy.

### Design and procedures

Approval for this study was obtained from the Institutional Review Board affiliated with the university overseeing this study. Data were collected via one‐on‐one interviews, and participants verbally responded to survey measures about housing and neighborhood experiences, mental health symptomatology and recovery, service use, and demographics. Surveys were administered by a team of four trained student research assistants, and answers were recorded electronically on iPads. Participants were informed of the risk of minimal discomfort from questions about sensitive issues before signing an informed consent document. Participants were provided a $20 cash incentive for participating. Interviews lasted between 1.5 and 2 h and were conducted in participants' homes or common areas on site.

### Measures

#### Housing staff support

The housing staff support scale is a composite measure created specifically for this study to capture residents' perceptions of support provided by housing staff. Staff support was operationalized as the residents' perception of their relationship with housing staff, the frequency of interactions, and their satisfaction with the assistance provided. This measure focuses on relational, tangible, and practical aspects of support in supportive housing environments. The scale consists of three items, each measured using a five‐point Likert scale. The first item, “How well do you and your housing staff know each other?” (1 = *Not at all* to 5 = *Very well*), assesses the degree of familiarity and relational closeness between residents and staff. The second item, “How much contact have you had with your housing staff in the past 6 months?” (1 = *Not at all to* 5 = *At least once a day*), reflects the frequency of interaction, which is a critical component of staff support. The third item, “Overall, how satisfied are you with your housing staff?” (1 =* Very dissatisfied* to 5 = *Very satisfied*), captures residents' overall satisfaction with housing staff, reflecting their perceptions of the staff's helpfulness and effectiveness.

The decision to include these items was guided by the need to operationalize staff support as a multi‐dimensional construct encompassing relationship quality, contact frequency, and satisfaction. These elements align with existing literature on social and practical support, which emphasizes the importance of trust, familiarity, and perceived helpfulness in fostering positive outcomes. Together, these items were designed to capture both the relational and practical aspects of housing staff support that are central to the supportive housing environment. The internal reliability (i.e., Cronbach's *α*) for the scale was 0.54, which is low but argued to be adequate for an exploratory field‐based study in the social sciences, particularly for scales with a small number of items, such as this one (Hair et al., 2006).

#### Loneliness

Loneliness was measured using a four‐item version of the UCLA Loneliness Scale (Russell et al., 1980). Each item was rated on a four‐point scale from never to always and asked how often individuals felt left out; that there are people that really understand them; isolated from others; and that there are people they can talk to. The original UCLA Loneliness Scale demonstrates good validity and reliability (Russell, 1996; Russell et al., 1980). The Cronbach *α* in this study was 0.76, which is acceptable.

#### Residential satisfaction

Residents' satisfaction with their housing and neighborhoods was measured using the Housing Environment Scale–Resident Satisfaction subscale (HES‐RS; Wright & Kloos, 2007). Participants answered four questions, two using a five‐point Likert scale and two using a three‐point scale, to evaluate satisfaction and comparison to previous living situations. The scores were rescaled for a composite score, resulting in a Cronbach's *α* of 0.65, indicating adequate reliability.

#### Sense of community

Sense of community was measured using the Sense of Community Index‐2 (McMillan & Chavis, 1986). Participants answered 24 items assessing perceptions of community membership, influence, fulfillment of needs, and shared emotional connection (e.g., “Members of this community care about each other” and “If there is a problem in this community, members can get it solved”). Items were rated on a 4‐point Likert scale ranging from (1) *Not at All* to (4) *Completely*. The Cronbach *α* for the scale in this study was 0.94, which is very good.

#### Self‐determination

Deci and Ryan (1985) suggest that there are three integral components of self‐determination: autonomy, competence, and relatedness. To assess each of these components, three subscales from Ryff's Psychological Well‐Being short‐form scale (Ryff & Keyes, 1996) were used, which has been recommended by others in this literature (e.g., Gao & McLellan, 2018). An example item is “I have a sense of direction and purpose in my life” (Ryff & Keyes, 1996). The three‐item autonomy subscale was used to capture the autonomy component of self‐determination. The Cronbach *α* for this nine‐item scale was 0.74, which is acceptable.

#### Brief Symptom Inventory (BSI)

The BSI (Derogatis, 1993) was used as a covariate in the analysis to control for psychiatric symptom distress. The BSI is a 53‐item self‐report measure that assesses psychological distress across nine primary symptom dimensions, including depression, anxiety, somatization, and interpersonal sensitivity, as well as providing an overall measure of distress. Participants rated the severity of their symptoms over the past week on a 5‐point Likert scale ranging from 0 (*not at all*) to 4 (*extremely*), reflecting current levels of psychological distress. An example item is “Feeling that you are watched or talked about by others.” The Cronbach *α* for this scale in this study was 0.96, which is very good. The inclusion of the BSI as a covariate allows for the control of participants' current psychological distress, ensuring that the analysis isolates the effects of housing staff support on well‐being.

### Analysis

All statistical analyses were conducted using IBM SPSS version 28. Data were screened for outliers and data entry errors before conducting analyses. Listwise deletion was used for participants missing more than 15% of the data on any variable (i.e., requiring a completion rate of 85% or higher for inclusion in analyses). No appreciable amount of data was missing, so no participants were excluded (Table 1).

**Table 1:** Descriptive statistics.

Measure	*N*	Min	Max	Mean	SD	Skewness		Kurtosis	
						Statistic	SE	Statistic	SE
Housing staff support	172	1	5	3.51	0.76	−0.52	0.19	0.03	0.37
Loneliness	169	1	4	2.16	0.72	0.21	0.19	−0.31	0.37
Residential satisfaction	172	1	3	2.65	0.39	−1.52	0.19	2.54	0.37
Sense of community	171	1	3.92	2.42	0.63	0.16	0.19	−0.37	0.37
Self‐determination	168	2.11	5	3.65	0.58	−0.05	0.19	−0.19	0.37

To address the study's research questions, the analysis followed two main steps. First, a series of *t*‐tests, analysis of variances, and bivariate correlations were conducted as preliminary analyses to identify significant covariates (see Table 2 for correlation matrix of study variables). Gender and psychiatric symptom distress emerged as significant covariates and were controlled for in subsequent analyses. Race and age were not significant covariates and were excluded from subsequent analyses for the sake of model parsimony and statistical power.

**Table 2:** Correlation matrix of study variables.

	Housing staff support	Loneliness	Residential satisfaction	Sense of community	Self‐determination
Housing staff support	‐‐				
Loneliness	−0.23*				
Residential satisfaction	0.19**	−0.00			
Sense of community	0.44*	−0.29*	0.18**		
Self‐determination	0.23*	−0.52*	0.07	0.17**	‐‐

* Correlation is significant at the 0.01 level (2‐tailed).

** Correlation is significant at the 0.05 level (2‐tailed).

For Research Question 1, three separate multiple regressions were conducted to examine the relationships between housing staff support and resident experiences (loneliness, residential satisfaction, and sense of community), while controlling for the identified covariates.

For Research Question 2, moderated regression analyses were conducted to test whether self‐determination moderated the relationships between housing staff support and loneliness, residential satisfaction, and sense of community. The moderation analyses explored the conditional effects of housing staff support at varying levels of self‐determination (one standard deviation below the mean, at the mean, and one standard deviation above the mean). This structured approach allowed for testing both direct relationships and potential interaction effects.

Moderation models were selected to explore how self‐determination influences the relationship between staff support and resident experiences, as this approach aligns with the study's focus on identifying conditions under which staff support is most effective. Given the cross‐sectional nature of the data, moderation is particularly appropriate because it avoids assumptions about causal pathways inherent in mediation models, which require temporal sequencing. However, potential mediation pathways remain an important consideration and are discussed as a limitation and direction for future research.

## RESULTS

**Research Question 1:** Does housing staff support in a supportive housing environment relate to residents' loneliness, residential satisfaction, and sense of community?

Three multiple regressions were conducted to examine the relationship between housing staff support and each outcome after controlling for gender and psychiatric symptom distress. The first model testing the relationship between housing staff and loneliness was significant, *F* (3, 164) = 20.75, *p* < .001 and explained 26.8% of the variance in loneliness. Housing staff support was significantly negatively associated with loneliness (*B* = −0.15, SE = 0.07, CI [−0.29, 0.09], *p* < .05) and psychiatric symptom distress was significantly positively associated with loneliness (*B* = 0.51, SE = 0.08, CI [0.36, 0.66], *p* < .001), while gender was not significant. For residential satisfaction, the model with housing staff support and the covariates predicting residential satisfaction was significant, *F* (3, 164) = 5.3, *p *< .05, and explained 8.3% of the variance in residential satisfaction. Housing staff support was positively associated with residential satisfaction (*B* = 0.09, SE = 0.042, CI [−0.28, −0.04], *p* < .05), and gender was negatively associated with residential satisfaction (*B* = −0.16, SE = 0.06, CI [−0.14, 0.05], *p* < .01), indicating that female and nonbinary participants (grouped together because only one participant was nonbinary, and they disclosed being assigned female at birth) had lower residential satisfaction than male participants (*B* = −0.16, SE = 0.06, *p* < .01). Psychiatric symptom distress was not significantly associated with residential satisfaction in this model.

Finally, for sense of community, the model with housing staff support and covariates predicting SOC was significant, *F* (3, 164) = 14.65, *p* < .01, and explained 20% of the variance in sense of community. Housing staff support was significantly positively associated with residents' sense of community (*B* = 0.38, SE = 0.061, CI [0.26, 0.50], *p* < .01), while neither of the covariates were significant.

**Research Question 2**: How does self‐determination affect the strength of the association between housing staff support and residents' loneliness, residential satisfaction, and sense of community?

A moderated regression model was tested to investigate whether the association between staff support and the three constructs (i.e., loneliness, residential satisfaction, and sense of community) depends on the resident's level of self‐determination. The first moderated regression model investigated whether the association between staff support and loneliness depends on the client's level of self‐determination. The interaction between housing staff support and self‐determination was not significant (*B* = 0.02, SE = 0.12, CI [−0.19, 0.23] *p* = .85). Increased staff support related to decreased loneliness regardless of the amount of self‐determination.

The second moderated regression model tested whether the relationship between housing staff support and residential satisfaction depends on the resident's level of self‐determination. As hypothesized, the interaction between self‐determination and staff support was significant (*B* = −0.17, SE = 0.07, CI [−0.32, −0.04], *p* < .05). The interaction was investigated further by testing the conditional effects of housing staff support at three levels of self‐determination, one standard deviation below the mean, at the mean, and one standard deviation above the mean. Staff support was significantly related to residential satisfaction when self‐determination was one standard deviation below the mean (*B* = 0.19, SE = 0.06, CI [0.08, 0.30], *p* < .01) and at the mean (*B* = 0.08, SE = 0.04, CI [0.00, 0.17], *p* < .05), but not when self‐determination was one standard deviation above the mean (*B* = 0.02, SE = 0.05, CI [−0.14, 0.10], *p* = .74). The relationship between housing staff support and residential satisfaction was significant when self‐determination was less than 0.45 standard deviations above the mean but not significant with higher levels of self‐determination.

The final moderated regression model investigated whether the association between staff support and sense of community depends on the resident's level of self‐determination. The interaction between housing staff support and self‐determination was not significant (*B* = 0.04, SE = 0.10, CI [−0.16, 0.24], *p* = .69). Increased staff support related to increased sense of community regardless of the amount of self‐determination.

## DISCUSSION

### Relationship between staff support and resident experiences

Utilizing the social ecological approach to investigate housing programs suggested by Kloos and Shah (2009), the primary purpose of this study was to better understand the relationship between staff support and resident experiences of loneliness (interpersonal), residential satisfaction (housing and neighborhood), and sense of community (community). As hypothesized, results indicated that there was a significant negative relationship between staff support and loneliness as well as a significant positive relationship between staff support and both residential satisfaction and sense of community. These findings suggest that staff may be able to support residents beyond clinical and housing outcomes alone, and indeed they may be an essential component of the supportive housing environment. While future research is needed to identify the causal mechanisms driving the relationship between staff support and resident outcomes, the existing literature points to a few potential explanations for this association.

Staff support may relate to decreased loneliness simply by increasing the number of weekly social interactions available to residents, particularly for those who may have limited social networks. Residents may have few social interactions outside of their service providers, so small yet consistent social exchanges with housing staff in the hallways or on‐site offices could influence reducing loneliness. This reasoning would be consistent with literature of populations in the medical field, which found that patients who require more staff support with daily living activities reported lower levels of loneliness, even after controlling for social network quality (Franklin & Tranter, 2006).

Staff support was also found to relate to increased residential satisfaction, which assesses how satisfied the individual is with their housing and neighborhood and how this compares to their previous living situation. One possible reason for this association may be that housing staff are able to assist residents in improving the physical conditions of their living situation. For instance, a resident may need help fixing a leaky sink and reach out to their housing staff for support. Thus, an increase in staff support may result in a better physical quality of one's living space and therefore a higher satisfaction. One qualitative study supports this idea and found that staff were important in helping individuals in a supportive housing address both practical concerns as well as to mediate issues with other tenants (Bengtsson‐Tops et al., 2014). Further, previous studies have shown that supportive housing staff can assist residents with both case management concerns and connecting residents with community programs and resources (Forenza & Lardier, 2017; McCarthy & Nelson, 1993).

### The moderating role of self‐determination

The moderated regression analyses found that self‐determination did not moderate the relationship between staff support and loneliness or sense of community. It was hypothesized that motivated residents would benefit more from staff support, but findings revealed staff support was associated with decreased loneliness and increased sense of community regardless of self‐determination levels. One possible explanation for these nonsignificant findings could be the nature of the social and emotional aspects of staff support. Loneliness and sense of community are relational constructs that may be influenced more by the frequency and quality of interactions with staff than by residents' levels of self‐determination. Residents may benefit from low‐pressure, informal social connections with staff regardless of their intrinsic motivation or perceived autonomy. Additionally, loneliness and sense of community may reflect experiences of connection that are not contingent on personal agency but rather on the availability and consistency of staff presence. This aligns with prior research showing that reliable, supportive relationships are critical for fostering social connectedness among individuals with serious mental illness, irrespective of individual‐level characteristics (Townley & Kloos, 2009; Yanos, 2007).

Interestingly, self‐determination did moderate the relationship between staff support and residential satisfaction. For residents with low to moderate self‐determination, staff support had a stronger positive association with residential satisfaction compared to those with high self‐determination. Highly self‐determined individuals did not significantly benefit from staff support in terms of residential satisfaction, indicating that staff support may be more important for those with lower self‐determination.

As mentioned previously, staff provide a wide range of support in the supportive housing setting. Some such supports include addressing practical concerns like maintenance requests, keeping the property clean, and addressing any safety concerns. The findings from the study may suggest that these types of support may be more important for those who are low or moderate in self‐determination. Those who are highly self‐determined may find it easier to take initiative and improve their physical conditions, rather than needing staff to help them do this. Those with lower levels of self‐determination may benefit greatly from the assistance of staff in addressing concerns about their living environment.

These findings suggest that self‐determination may play a role in how residents experience and respond to different supportive housing models. While the moderation effect was only found for residential satisfaction, this raises questions about whether individuals with higher levels of self‐determination might prefer housing environments that offer more autonomy and less structured support. For instance, scattered‐site housing—where residents live independently and access services as needed—may align better with the preferences or strengths of more self‐determined individuals compared to congregate or single‐site models with more on‐site structure. Although these possibilities were not directly tested in this study, future research could explore whether self‐determination influences which types of supportive housing are most beneficial for different individuals. Understanding this relationship may help inform more person‐centered housing placements and support strategies.

The overall finding of the moderation analysis suggests that regardless of the level of self‐determination, staff support was still associated with favorable resident experiences. While this is different from what was hypothesized, these findings show promise that staff support is associated with wellbeing for individuals at different stages of recovery—not just those who are high in autonomy, competence, and relatedness.

### Limitations

Before discussing the study's implications, several limitations should be acknowledged. The study's localized sample from a specific housing program limits generalizability to other programs and locations. Additionally, the data were collected ten years ago, which may not reflect the current context, especially considering the impacts of the COVID‐19 pandemic on supports and services for people with SMI (Moreno et al., 2020).

Response bias is another limitation, as participants were interviewed one‐on‐one, which might have led to overreporting positive perceptions and underreporting negative ones. Despite assurances of confidentiality, concerns about how negative responses could affect their housing or services may have influenced participants' answers. Fluctuations in psychiatric symptoms could also affect responses, though the study accounted for this by including psychiatric symptom distress as a covariate. However, this may not fully capture daily symptom variations. The cross‐sectional nature of the study prevents causal inferences; lower loneliness, for instance, might be due to participants' extroversion rather than staff support.

The staff support measure's low inter‐item reliability, likely due to the small number of items, suggests it may not fully capture the breadth of staff support. Future research should refine this scale to better represent the construct. Finally, the potential for data clustering from participants across 16 supportive housing sites suggests a need for hierarchical linear modeling (HLM). Although this study lacked sufficient power for HLM, future research should aim for larger samples across more sites to address potential systematic differences.

While this study focused on moderation analyses to explore how self‐determination influences the relationship between staff support and resident experiences, it is possible that self‐determination may also serve as a mediator in this relationship. Mediation models, which examine how or why relationships occur, could provide additional insights into the mechanisms through which staff support influences resident experiences. However, the cross‐sectional nature of the data limits causal interpretations, and future research utilizing longitudinal designs would be better suited to test these pathways.

### Implications for practice

The information gained in this study has widespread implications for researchers, policymakers, and perhaps most importantly, the residents and staff of supportive housing programs. This study focuses on one of the most critical features of supportive housing—the support of on‐site staff. One of the predominant criticisms of supportive housing is that residents are not receiving the support they need to be successful in these programs (Padgett, 2013). This study found that staff support is indeed related to lower levels of loneliness, higher residential satisfaction, and a higher sense of community. Research examining staff support is essential in showing policymakers and funding agencies that these programs go beyond simply housing individuals.

While the role and value of staff support in supportive housing should continue to be explored, findings of this study suggest the importance of allocating funding towards the continued presence and availability of on‐site staff support. Further, programs can use this information to develop enhanced staff training to ensure that they adequately support the needs of their residents. Moreover, housing researchers, advocates, and policymakers can use these findings to demonstrate the presence and value of staff support in supportive housing environments, thus challenging assertions that individuals are not being adequately supported to be successful in housing.

Findings from this study contribute to this discussion by highlighting the importance of housing staff support in reduced loneliness, increased sense of community, and greater residential satisfaction, particularly for residents with low to moderate levels of self‐determination. These findings suggest that staff play a crucial role in improving experiences of residents by addressing practical concerns, facilitating social connections, and supporting residents in navigating challenges within their living environment. However, the nonsignificant moderation effects for loneliness and sense of community highlight a potential limitation: staff support alone may not fully address residents' needs for social connectedness or broader community engagement, particularly in the absence of more structured programming or peer involvement.

To address these critiques, supportive housing programs may benefit from improving the consistency and personalization of staff support. Ensuring that staff are adequately trained to recognize and respond to residents' varying levels of self‐determination may help optimize the impact of their support. Additionally, implementing resident‐driven programming and increasing opportunities for peer‐led initiatives may help bridge gaps in social and community integration that are not addressed solely through staff assistance. By addressing these critiques and tailoring supportive housing services to better meet residents' needs, programs can further their goals of fostering independence, well‐being, and meaningful community participation for individuals with serious mental illnesses.

### Implications for research

Existing research in supportive housing primarily centers on housing‐related outcomes rather than focusing on overall well‐being and resident experiences (Aubry et al., 2020). While this study is essential in demonstrating baseline‐level effectiveness of such programs, this only captures one small piece of an individual's experience. Supportive housing is intended to not just house individuals with serious mental illnesses, but also to allow them to thrive as functioning members of their communities. Research on supportive housing should reflect these goals to demonstrate the program's effectiveness beyond only housing retention alone. This study contributes to the limited research on loneliness, residential satisfaction, and sense of community among residents of supportive housing.

Future research can build upon the findings of this study by exploring the role of staff support in other regions of the country and internationally to see if this association expands beyond the local context. As previously mentioned, COVID‐19 has drastically impacted the ways programs are run and more broadly how individuals relate to one another. Some supportive housing programs have begun offering their services remotely in response to the pandemic. For instance, instead of having on‐site staff support residents may have the option to schedule phone or video appointments with their housing staff. It would be interesting to see if these remote services are comparable to the in‐person services that were examined in this study. Is all staff support helpful to residents or is there something inherently unique about in‐person interactions that allow staff support to have a positive impact?

Further, the staff support scale used in this study was a composite scale created for the purposes of this study and may not fully capture the scope of services being provided by staff. Future research could engage in further scale development to better capture the concept of staff support and identify what specific components of staff support are influential. Moreover, a mixed‐methods design could add more depth to these findings to better understand this phenomenon from both the resident and staff perspective. For instance, what type of interaction with their staff makes residents feel most supported?

Finally, this study contributes to the limited research utilizing a social‐ecological approach to understanding individuals' experiences in supportive housing. Capturing the complexities of the human experience in an ever‐changing environment is a demanding task, particularly when using cross‐sectional quantitative methodology that may not adequately reflect the myriad contextual factors at play. An ecological approach allows for a more in‐depth understanding of an individual's experience. This study provides a framework for future researchers to better understand individuals' experiences in supportive housing, and the important role that housing staff may play in their housing success and broad experiences of thriving.

## CONFLICT OF INTEREST STATEMENT

The authors declare no conflicts of interest.

## Data Availability

The participants of this study did not give written consent for their data to be shared publicly, so due to the sensitive nature of the research supporting data is not available.
